# Insights into flagellar function and mechanism from the squid–vibrio symbiosis

**DOI:** 10.1038/s41522-019-0106-5

**Published:** 2019-10-25

**Authors:** Marie-Stephanie Aschtgen, Caitlin A. Brennan, Kiel Nikolakakis, Stephanie Cohen, Margaret McFall-Ngai, Edward G. Ruby

**Affiliations:** 1 Department of Medical Microbiology and Immunology, University of Wisconsin-Madison, Madison, WI 53706 USA; 2 Laboratory for Biological Geochemistry, School of Architecture, Civil and Environmental Engineering, Ecole Polytechnique Fédérale de Lausanne, and Center for Advanced Surface Analysis, Institute of Earth Sciences, Université de Lausanne, CH-1015 Lausanne, Switzerland; 3 Kewalo Marine Laboratory, University of Hawaii-Manoa, Honolulu, HI 96813 USA; 4 Present Address: Department of Microbiology, Tumor and Cell Biology, Karolinska Institute, Solna, 171 76 Sweden; 5 Present Address: Department of Immunology and Infectious Diseases, Harvard T.H. Chan School of Public Health, Boston, MA 02115 USA; 6 Present Address: Department of Natural and Applied Sciences, University of Wisconsin – Green Bay, Green Bay, WI 54311 USA

**Keywords:** Cellular microbiology, Symbiosis

## Abstract

Flagella are essential and multifunctional nanomachines that not only move symbionts towards their tissue colonization site, but also play multiple roles in communicating with the host. Thus, untangling the activities of flagella in reaching, interacting, and signaling the host, as well as in biofilm formation and the establishment of a persistent colonization, is a complex problem. The squid–vibrio system offers a unique model to study the many ways that bacterial flagella can influence a beneficial association and, generally, other bacteria–host interactions. *Vibrio fischeri* is a bioluminescent bacterium that colonizes the Hawaiian bobtail squid, *Euprymna scolopes*. Over the last 15 years, the structure, assembly, and functions of *V. fischeri* flagella, including not only motility and chemotaxis, but also biofilm formation and symbiotic signaling, have been revealed. Here we discuss these discoveries in the perspective of other host–bacteria interactions.

## Introduction

The roles of bacterial flagella and motility in pathogenic host–microbe interactions have long been studied.^1,2^ Over the last decade, the increased interest in beneficial symbioses have revealed both similar and novel flagellar structures and functions, albeit underlying quite different biological outcomes. The features of certain symbiotic systems have made them models for studying specific aspects of host–bacteria interactions. For example, the symbiosis between the bioluminescent *Vibrio fischeri* and the Hawaiian bobtail squid, *Euprymna scolopes*, is an excellent model in which to study the role of flagella in the initiation and persistence of colonization. Features of this symbiosis have also contributed to the study of the flagellum’s structural complexity, and helped pinpoint exactly when and where flagella-related functions are required during the interaction.

**Fig. 1:**
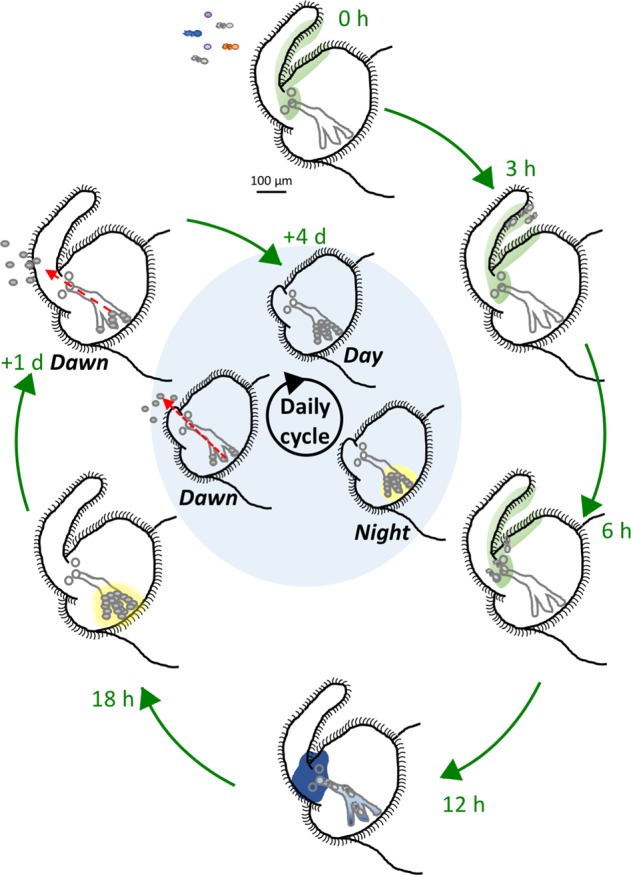
‘Clock’ illustrating the temporal unfolding of initiation events during the first 4 days of symbiosis. Within 0.5 h of hatching, the host’s ciliated field responds to exposure to ambient bacterial PGN by shedding mucus (light green) in which *V. fischeri* (gray, flagellated cells) can attach, and then aggregate. Within 4–6 h, the aggregate migrates to the pores, and uses swimming motility and chemotaxis to follow a chemoattractant gradient (dark blue) to enter the surface pores, reaching the deep crypts by between 7 and 10 h. By 12–18 h, the bacteria have multiplied, filling the crypts and inducing bioluminescence (yellow). At dawn, 95% of the bacteria are expelled (red arrow) and the remaining 5% proliferate, starting a new day/night cycle. The presence of the symbionts triggers the development of the light organ, including the full regression of the ciliated field and appendages over the first four days

*V. fischeri* specifically colonizes the dedicated light-emitting organ of sepiolid squids (Fig. 1; Box 1; for review, see McFall-Ngai, 2014).^3^ The exact benefits of this symbiosis are still unknown but people have speculated that the bioluminescence produced by *V. fischeri* provides the squid with a camouflage mechanism. The light produced is similar in intensity and color to moonlight and may prevent the squid from casting a shadow during its nocturnal hunting. In exchange, the bacteria are provided with a nutrient-rich niche with no competition (Box 1). This mutualism starts when the newly hatched, aposymbiotic squid recruits *V. fischeri* cells from the surrounding seawater environment. This recruitment process requires *V. fischeri* to migrate through mucus, from aggregates assembled at the surface of the light organ, through pores, and on into crypts deep within the tissue.

**Fig. 2:**
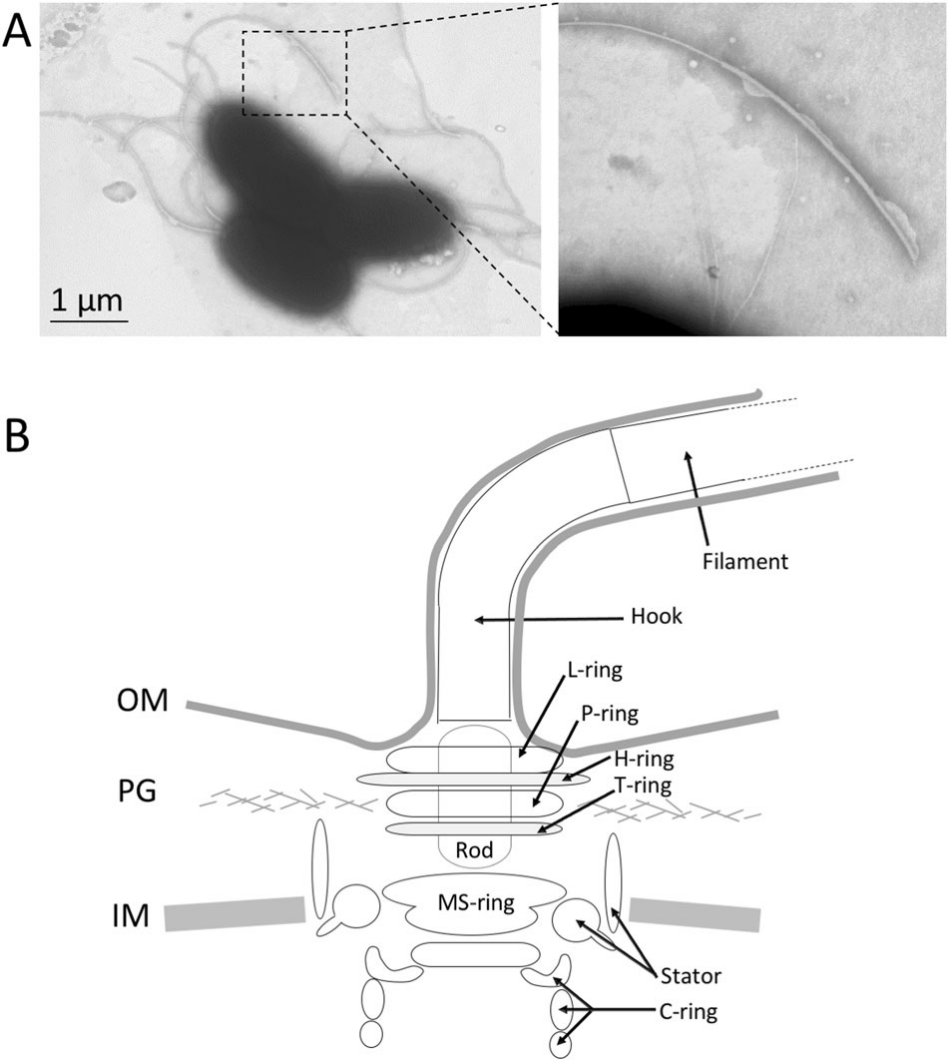
Flagellar structure of *V. fischeri*. **a** Electron-microcopy image of the tuft of polar flagella of *V. fischeri* (**b**) Schematic diagram of the hook and basal body of a polar flagellum. The bacterial flagellum consists of three parts: the filament, the hook and the complex basal body embedded in the outer membrane (OM), inner membrane (IM), and peptidoglycan (PG). The rings present in the basal body that are specific to *Vibrio* spp. (T-ring and H-ring) are indicated in green, and the OM-derived sheath is depicted in dark gray

The motility apparatus of *V. fischeri* cells is a polar tuft of 2–7 sheathed flagella (Fig. 2a), which are present on planktonic cells as they initiate colonization, but are not detected on cells within the crypts.^4^ The flagella are essential to reach the deep crypts,^5,6^ and also provide a key mechanism for signaling during symbiosis initiation. Indeed, both the flagella themselves and the motility they provide play a role in chemotaxis, biofilm formation, and the induction of host development, all of which are required to establish a sustained symbiosis.^7,8^ The tuft of lophotrichous flagella of *V. fischeri* is an uncommon feature among bacteria. Among the *Vibrio* spp.^9^, this feature is shared only by its most closely related congeners, *V. logei* and *V. salmonicida*. In addition, flagella of *V. fischeri* are surrounded by an outer membrane-derived sheath,^6^ another unusual feature of bacterial flagella, present in only a few other genera, such as *Bdellovibrio*, *Helicobacter*, and *Brucella*.^10^ The squid–vibrio model offers an opportunity to study the functions of these flagellar features, as well as the role of flagella in the different steps of host colonization, providing insights into the function of this cellular apparatus in the establishment of symbiosis in general (Box 2).

In this review, we use events in the initiation of the squid–vibrio symbiosis not only to discuss new findings on the structure and assembly of the flagella of *V. fischeri*, but also to describe how flagella more generally impact microbial behaviors, including facets of host interaction. We specifically chose to highlight features that are either shared with other symbiotic or pathogenic relationships, or are elements apparently specific to the squid–vibrio model.

Box 1 The squid–vibrio symbiosis *Vibrio fischeri* specifically colonizes the dedicated light-emitting organ of sepiolid squids.^120,123^ This mutualism starts when the newly hatched, aposymbiotic squid recruits *V. fischeri* cells from the surrounding seawater environment, a process that can be divided into three steps: (i) the initiation phase, which occurs during the first 12 h, (ii) the accommodation phase, extending over the next few days, as the symbiosis develops and matures, and (iii) the persistence phase, during which the mature symbiotic state is achieved and maintained. *V. fischeri* present in the bacterioplankton are ventilated into and through the squid’s mantle cavity,^29^ and as few as five cells, aggregating on the external ciliated surface of the nascent light organ^60^ will lead to a successful initiation (Fig. 1). Most, but not all,^2^ strains will pause 2–3 h in this aggregate, after which the bacteria migrate into surface pores, down ducts, and through an antechamber and a restrictive bottleneck. On average, one colonizing bacterium passes the bottleneck and reaches the deep epithelium-lined crypts,^72,124^ in which the resulting population of several hundred-thousand luminous symbionts derived from this sole colonizer is housed. The accommodation phase is initiated by the dawn light cue following the first night of colonization. As the symbionts reach high density in the crypts, levels of microbe-associated molecular patterns (MAMPs),^92^ such as peptidoglycan (PGN) and lipopolysaccharide (LPS) increase. The presence of these MAMPs, together with the bioluminescence of the symbionts,^125^ triggers many of the reversible and irreversible steps in the morphogenesis of the light organ.^120^ Finally, during its persistence in the developmentally mature light organ, the symbiont population is subject to, and maintained by, a dynamic daily rhythm imposed by the host.^82,126,127^ Each day, the dawn light cue induces the squid to expel most of its bacterial population into the surrounding seawater.^5^ The remaining symbionts rapidly multiply, reaching their typical high density by about noon,^107,128^ and the organ increases its light production soon after dusk (Fig. 1). This cycle is repeated throughout the lifetime of the host, and ensures the presence of a healthy, active symbiont population each night.

Box 2 The squid–vibrio system provides a good model to study symbiosis mechanisms During the last decade, technologies such as whole-genome sequencing have allowed researchers to characterize symbiotic communities more fully, and to highlight the fundamental role of the microbiota in every activity of living organisms. However, faced with the complexity typical of such microbial communities in most animals, scientists are developing and using simpler model systems with which to more easily study the fundamental mechanisms governing symbiotic associations. The binary association between *Euprymna scolopes* and *Vibrio fischeri* provides a rare opportunity to study the initial steps in the colonization of animal epithelia, the most common of symbiotic interactions. In addition to the availability of the sequenced genomes of both partners (*Vibrio fischeri:* NC_006840.2; NC_006841.2 NC_006842.2 and ^70^and *Euprymna scolopes*: BioProject PRJNA470951^105^), many genetics tools have been developed in *V. fischeri* and, because the symbionts are transferred horizontally at each generation, experimental manipulation of the initiation of the association is simplified. Similarly, the development of advanced imaging approaches^29,120^ has made it possible to localize not only individual migrating symbiont cells, but also the host responses they induce along the colonization pathway, clarifying key events. The initial steps of the squid colonization have been well described and are subject to experimental manipulation, especially because the fitness of an aposymbiotic animal is not compromised in the laboratory. Taken together, with the available bacterial genetic toolkit, these characteristics have allowed a dissection of the different functions of the symbiont’s flagellum by following the bacterium in real time using microscopy, and to document their progression through the checkpoints of initiation.

## The flagellar machinery of *Vibrio fischeri*

*Structure of the flagellum* (for review, see Erhardt, Namba and Hughes, 2010^11^).

Despite broad differences in their swimming capacity, the mechanism underlying motility is remarkably conserved among all flagellated bacteria. While the ion whose membrane gradient is used to power rotation (i.e., the proton- or sodium-motive force) and some protein components of the motor are species-specific, overall, the flagellum is composed of ~19 core proteins.^11^ Those core components are organized into three sub-complexes, assembled in the following order: (i) the rotor complex, (ii) the hook, and (iii) the filament (Fig. 2b). The rotor complex consists of the basal body, which anchors the machinery to the cell envelope, and is linked to the cytoplasmic C-ring, which provides the energy required to rotate the flagellum. The hook is a universal joint that connects the basal body to the filament, and is about 50 nm in length. Finally, the hook extends into a rigid protein-helix filament, which projects several micrometers from the cell body and functions as a propeller. The basal body has the most complex composition, consisting of several structures. The rod passes through the L- and P- rings, which are embedded in the outer membrane and peptidoglycan layer respectively, and likely anchor the flagellum to these layers. The MS-ring is located within and above the inner membrane. These four rings act as bushings that support the rod, which serves as a drive shaft spanning the inner and outer membrane.^12^ The MS-ring is part of the rotor complex and functions as an assembly platform for the other components. The C-ring is composed of the three switch proteins (FliG, FliM and FliN) and controls the direction of flagellar rotation. The flagellar motor converts the potential energy present in a transmembrane electrochemical gradient (sodium ion, in *Vibrio* spp.) into torque that is transmitted by the C-ring to the MS ring, then to the rod and from there to the hook, which rotates the filament.^11^ Depending on the number of flagella and their arrangement as either polar or peritrichous, different motility patterns are observed. In *Escherichia coli*, the peritrichous flagella form a single bundle only when rotating in the counterclockwise orientation, thereby propelling the cells forward. Changing the rotation to the clockwise direction disrupts the flagellar bundle, and leads to tumbling and reorientation of the cell. Those *Vibrio* spp. that have a single polar flagellum display a different swimming pattern, often described as push and pull. When the flagellum rotates in a counterclockwise direction, the bacterium is pushed forward. When the direction is reversed, the cell is pulled backward.^13^ However, this pattern has not yet been confirmed for *Vibrio* spp. with a tuft of polar flagella. Interestingly, in *Pseudomonas putida*, a third swimming behavior was described. This bacterium also has a polar tuft of flagella and can propel itself with its helical bundle wrapped around the cell body. This last pattern is believed to enhance their swimming capacity, especially when in contact with a surface.^14^ This pattern was also reported for *V. fischeri*;^15^ however, it has not yet been documented to occur when the bacterium migrates within the host.

While in most bacteria the flagellum filament is reported to be composed of one type of flagellin protein, in *Vibrio* spp. the filament can be comprised of up to six distinct flagellins.^9^ However, the potentially distinct functions of these additional types of flagellins are not yet elucidated. For example, in *V. cholerae* and *V. anguillarum*, only the flagellin FlaA is essential for motility and the assembly of a full-length flagellum.^16–18^ *V. fischeri* encodes six flagellins organized in two loci. The first locus encodes the five flagellin genes *flaABCDE*, and is localized near the other structural genes involved in motility and chemotaxis, while the second locus is distant and composed of only *flaF*.^19^ Although *V. fischeri* FlaA is homologous to the FlaA of other bacteria, the five other flagellins are unique and exhibit the greatest similarity to each other.^19^ This relationship, which is different from several other *Vibrio* spp. that have shared lineages of their *fla* operon, suggests that, whereas there might be functional constraints on the structure of the primary component, FlaA, the evolution of the other flagellins of *V. fischeri* has been independent and species (or clade) specific. In *V. fischeri*, FlaF is the only flagellin not found in the flagella filament in culture-grown cells.^19^ Similarly, in *V. vulnificus*, FlaE is absent from the filament.^16^ This latter result suggests that while these minor flagellins are not typically part of the filament, they may play a regulatory role or be assembled into the filament only under certain conditions, or for particular functions. For instance, such minor flagellins may play a role in host interaction; e.g., in *V. anguillarum*, FlaD and FlaE are essential for virulence in fishes,^20^ while in *V. salmonicida*, the loss of FlaD has only a modest effect on fish mortality.

Recently, it was suggested that some of the differences between the swimming abilities of bacteria result from either adaptation at the level of motor function or the spatial arrangement of several different flagellin proteins along the filament. For example, in *Shewanella putrefaciens*, the major flagellin is more abundant at the end of the filament closer to the motor, while a minor flagellin is more abundant along the rest of the filament. This discontinuous spatial arrangement of flagellins improves motility compared to when flagella are constructed of only the main flagellin.^21^ In *V. fischeri*, the arrangement of the flagellins in the filament is unknown, but the presence of an additional four minor flagellins in the filament could similarly contribute to the high velocity reported for this species. In addition to filament spatial organization, in the case of *Vibrio* spp., the sodium ion gradient-driven motor generates a torque between 2000 and 4000 pN nm, depending on the magnitude of the gradient,^22^ whereas the proton motors of *Salmonella enterica* generate a lower torque of between only 1300 and 2000 pN nm.^23^ The conserved core basal-body configuration of *Vibrio* spp. consists of two additional ring structures: (i) the T-ring, which assembles just beneath the P-ring and is formed by the MotX and MotY proteins, and (ii) the H-ring, composed of the *Vibrio*-specific proteins FlgT, FlgP, and FlgO, which stabilize the basal body.^24,25^ The H-ring is also involved in flagellar assembly by *Vibrio anguillarum*. Specifically, the H-ring facilitates the penetration (or extension) of the outer membrane by the filament: this ring’s absence results in the appearance of periplasmically located flagellar filaments that do not project into the environment.^26^ Thus, the H-ring may also play a role in the creation of the sheath, as the filament pokes into the membrane, projecting it outward.^26^ Further, these proteins have become essential motility components in other *Vibrio* spp.^24,27^. Based on these observations, a recent study revealed that *V. fischeri* incorporates 13 stator complexes compared to the 11 complexes typical of enteric bacteria.^28^ This wider rotor, along with the presence of the additional H-ring, is believed to explain the higher torque produced by *V. fischeri* motors, and may confer a more effective swimming capacity and speed in high-velocity and/or high-viscosity fluid environments such as those created by the ciliated field surrounding the *E. scolopes* light organ.^29^

### Regulation of flagellar assembly

About 50 genes are involved in the elaboration of a functional flagellum,^30^ which is a multi-step process with several checkpoints. In general, the proteins composing the structure of the basal body are expressed and assembled first, followed by construction of the hook and, finally, the filament (Fig. 2). Any defect in producing the hook will prevent the energetically expensive filament production and assembly from proceeding. To ensure the proper assembly order, bacteria have evolved finely regulated expression cascades. For instance, in *V. fischeri*, the genes of the flagellar operon, which is encoded on the large chromosome,^31^ are expressed in a manner similar to that of the well described regulatory cascade in *V. cholerae*.^27,32–34^ Specifically, flagellar-gene regulation occurs through a four-level cascade.^7^ The cascade is controlled first by the σ^54^-dependent, flagella master regulator, FlrA, which activates the early flagellar genes, such as those encoding the 2-component regulator FlrBC and σ^28^ (FliA). The late genes, encoding the filament and the motor proteins, are expressed in a sequential manner by FlrBC and, subsequently, σ^28^; interestingly, while *flaA* is under the control of FlrC, the minor flagellin genes seem to be regulated by the σ^28^ factor.^19,35^

Regarding the regulation of the number of flagella assembled at the pole, the tuft of 2–7 flagella elaborated by the fischeri clade^36^ is unique. Most other *Vibrio* spp. bear a single polar flagellum, while any additional peritrichous flagella are encoded by a heterologous operon located on the small chromosome.^37^ In *V. alginolyticus*, two antagonist proteins, FlhF and FlhG, either positively or negatively regulate the flagella number, respectively.^38,39^ Deletion of *flhG* in various species, including *Pseudomonas aeruginosa*, *V. cholerae* and *Campylobacter jejuni*, causes hyperflagellation.^39–41^ In *V. fischeri*, spontaneous hyperswimmer (HS) mutants assemble up to 15 polar flagella.^4^ However, whole-genome sequencing of two different classes of HS mutants failed to identify an underlying mutation(s). It seems that in *V. fischeri*, the number of polar flagella might be regulated by posttranscriptional mechanisms,^42^ suggesting another, yet uncovered, system of flagella regulation in the genus *Vibrio*. FlhF and FlhG are also involved in the structure’s polar localization.^40^ Finally, mutation of a chaperone *dnaJ* family gene changes the flagellum placement from polar to peritrichous.^43^

An intimate link has been established between virulence and motility in many bacteria.^44^ Indeed, in some species, the regulators of flagellar production are also involved in the modulation of virulence genes. In the last 15 years, using the squid–vibrio model, a similar link between beneficial bacteria and their host has been found, in which both motility and colonization factors are regulated by the same pathway.^27,32,35^ For example, a gene homologous to *V. cholerae kefB* (VF_0220) is repressed in the *V. fischeri flrA* mutant.^32^ In *E. coli*, KefB protects cells from toxic metabolites; KefB might provide symbiotic *V. fischeri* cells with a similar protection from host-derived toxins in the light organ.^45^

## Swimming motility and squid colonization

The roles played by the several different modes of surface motility in the establishment of symbiosis are largely unknown. Most bacterial symbioses are initiated in the early stages of the host’s life, as juveniles acquire proper bacteria either from the environment or directly from the parent;^46^ thus, one of the first challenges to successfully establishing such an association is the capacity of the symbiont to reach its target tissue. As in the squid–vibrio system,^6^ in many bacteria–host interactions, flagellar motility is essential to successful tissue tropism and host colonization.^6,47–50^ However, the specific time and location at which flagellar motility is required are often not well characterized. In the squid–vibrio system, it is possible to manipulate the initial colonization steps and to separate flagellar swimming behavior from the other roles of the flagella. Hence, several studies have shown that swimming motility itself is essential for *V. fischeri* to colonize the light organ.^6,27,32–34^ Surprisingly, motility mutants can move from the aggregates to the pores of the light organ,^32^ perhaps with help from host ciliary activity.^29^ However, flagellar motility may be needed to migrate through the pores and successfully reach the deep crypts, where colonization occurs.^51^ After the symbionts reach the crypts, the flagellar genes are down-regulated, presumably because motility is not required for persistence in the light organ.^4^ Several mutations affecting the number of flagella assembled by *V. fischeri* or its swimming behavior and capacity, result in a decreased level of colonization.^4,19^ For example, in a *flaA* mutant, only 75% of the cells assemble a flagellum, and each cell bears fewer flagella, although they appear functional and exhibit a normal length.^33^ Further, the *flaA* mutant exhibits an overall delay in colonization, although it reaches the light-organ pores as quickly as wild type. Finally, in competition with the wild type, the *flaA* mutant is preferentially expelled from the light organ.^33^ This behavior suggests additional functions for *V. fischeri* flagella in host–symbiont interaction, such as reaching or attaching to sites in deeper, more secure, regions of the crypts.

In a wild-type colonization, most of the bacteria are aflagellate by 12 h after entering the light organ.^52^ A possible mechanism to explain this observation is the recent finding that polarly flagellated γ-proteobacteria, including *V. fischeri*, eject filaments under nutrient depletion.^53^ In contrast, the HS mutants remain flagellated after the initial colonization event, and proliferate to a diminished level.^4^ In pathogenic interactions, virulence factors are often expressed in a manner opposite to flagella.^54^ In the squid–vibrio symbiosis, the induction of bioluminescence through quorum signaling and the expression of the *lux* operon is essential to establishing a sustained colonization.^55,56^ One class of HS mutants exhibits a decrease in bioluminescence,^4^ although whether the two events are directly connected is still unknown. In many bacteria, the inhibition of motility promotes the development of a biofilm^57,58^ which, in *V. fischeri*, is important for symbiont recognition and symbiotic initiation.^59^ Thus, the overexpression of motility by the HS mutants might be predicted to repress genes required to establish the colonization. Another possibility is a direct link to the HS motility capacity. Because the HS mutants swim faster on solid medium, it is possible that these mutants are capable of quickly escaping the mucus secreted by the light-organ appendages (Fig. 1), thereby preventing the HS mutants from becoming properly primed^60^ or migrating normally to the light-organ pores^6^ (see below). In fact, HS mutants are defective for hemagglutination, tend to attach to the light-organ cilia less efficiently, and form smaller aggregates.^4^ More recent studies of the squid–vibrio model have shown not only that flagellar motility is essential to establishing the symbiosis, but also that the migration speed of the cells is also critical to competitive effectiveness.^2^ Future investigation of the squid–vibrio model offers a unique possibility to determine the effect of flagellar activity on colonization, a parameter that has not been explored in other bacteria–host associations.

## Chemotaxis and squid colonization

**Fig. 3:**
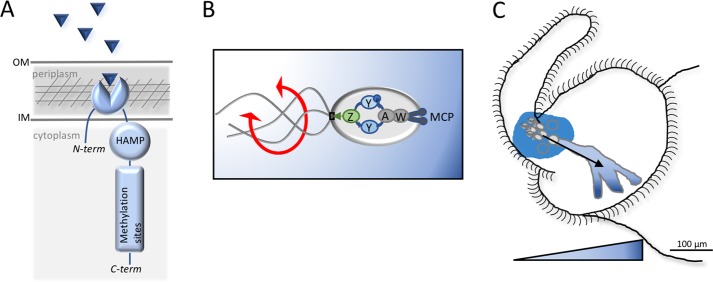
Chemotaxis in the squid–vibrio symbiosis. **a** Representative methyl-accepting chemotaxis protein (MCP) topology, including the ligand-binding domain, the HAMP domain, and methylation domain. Triangles represent potential ligands. **b** Simplified scheme of the chemotaxis signal-transduction cascade in *Vibrio* spp. Changes in chemoeffector levels are detected by the transmembrane MCP chemoreceptors. The resulting signal cascade starts with the SH3-like adaptor protein CheW, and passes to the CheA histidine kinase. In response to decreased chemoeffector concentration, the chemoreceptors activate CheA autophosphorylation. Phosphorylated CheA (CheA-P) phosphorylates CheY. CheY-P binds to the flagellar motor and promotes a switch in the direction of rotation from counterclockwise to clockwise. Finally, CheZ dephosphorylates CheY-P, allowing rapid termination of the signal response and re-setting of the pathway. In the absence of chemoeffector, CheA-P favors counterclockwise rotation. **c** Chemoeffectors secreted by the squid tissues establish a gradient (blue) extending out from the pores.^60,77^ *V. fischeri* cells are attracted by this gradient, and migrate through the pores to their final site of colonization, the deep crypts

In many associations, symbiont acquisition requires tissue tropism, i.e., active swimming toward the appropriate host tissue.^61^ Such an orientation of motility by bacteria is typically directed by a behavior called chemotaxis.^62^ By sensing gradients of attractants or repellents, bacteria can change their flagellar rotation, biasing the direction of their migration toward preferred environmental conditions. For instance, the nitrogen-fixing soybean symbiont *Bradyrhizobium diazoefficiens* encodes two flagellar systems; one of them, the lateral system, is up-regulated in the presence of a carbon source emanating from the plant that serves to guide the potential symbionts toward the roots.^63,64^ Such attractants (or repellents) are sensed by receptors called methyl-accepting chemotaxis proteins (MCPs). These sensory proteins are generally integral inner-membrane proteins that expose their ligand-binding domain in the periplasm and their signaling domain in the cytoplasm (Fig. 3a). Upon ligand binding, the MCPs undergo a conformational change, leading to the phosphorylation of their signaling domain. This step is followed by the autophosphorylation of the CheAY two-component system, which biases the rotational direction of the flagella^65^ (Fig. 3b). The process of chemotaxis and its component proteins have been best studied in *E. coli*, which encodes up to five MCPs involved in the sensing of amino acids, peptides, galactose, ribose, and oxygen.^66^ However, as more bacterial genomes have been sequenced, it has become apparent that bacterial chemosensing can be much more complex, with up to 61 distinct MCPs encoded by *Magnetospirillum magnetotacticum*, and a considerable structural variation between the MCPs of different species.^67,68^ It has been hypothesized that the complexity of a bacterial species’ MCP repertoire reflects the diversity of its lifestyle.^69^ The genome of the *V. fischeri* reference strain ES114 encodes one system of *che* genes and 43 putative MCPs,^70,71^ and closely related strains have a comparable number, but in distinctive sets.^72^ To date, only two MCPs have been characterized in *V. fischeri*, contributing to chemosensing of either amino acids, or short- and medium-chain length fatty acids.^71,73,74^ It is interesting to note that in less complex chemotaxis systems, such as in *E. coli*, all MCPs are part of the flagellar regulon;^75^ however, among the 43 MCPs of *V. fischeri*, only four are regulated by the flagellar master activator FlrA.^27^ This high diversity of MCPs likely reflects the necessity of *V. fischeri* to alternate between environmental conditions and niches (e.g., its planktonic and symbiotic lifestyles).^76^

In the squid–vibrio symbiosis, while chemotaxis is not absolutely essential for the symbiont, it strongly facilitates colonization efficiency.^34,71^ For example, under inoculation conditions that result in wild-type *V. fischeri* colonizing 100% of juvenile squid, a *cheY* mutant colonizes only about 50%. Not surprisingly, then, some signal(s) provided by the squid host appears to be a chemoattractant for *V. fischeri*.^77^ For instance, upon exposure to nascent symbionts, the light organ mediates the production of breakdown products of chitin (e.g., chitobiose and *N*-acetylglucosamine) and mucus *N*-acetylneuraminic acid (NANA),^78^ all of which are chemoattractants for *V. fischeri*.^79–81^ The nascent symbionts use the gradient of at least one of these products, chitobiose, to enter the pores and colonize the light organ.^77^ The squid-derived chitin-breakdown products are present in the light organ’s ducts and near the pores, creating a chemoattractant gradient leading *V. fischeri* cells into the deep crypts.^77^ To produce the chitin derivatives, which are also nutrients for its symbionts, the host expresses a chitinase whose expression follows the diel rhythm characteristic of the symbiosis.^82^ The host optimizes the symbiont’s chemotactic efficiency toward chitibiose even as *V. fischeri* cells are aggregated in the mucus surrounding the appendages. Specifically, when they are in the bacterioplankton, *V. fischeri* cells exhibit only a weak capability to sense and chemotax toward chitibiose (Fig. 3c). However, *E. scolopes* secretes a chitotriosidase (EsChitotriosidase) close to the light-organ pores, and in the mucus along the ciliated field of the appendages, creating a low concentration of chitin-breakdown products in the mucosal environment. This activity ‘primes’ the symbionts, enhancing by 4-fold their capacity to detect and swim toward the chitin gradient;^60^ thus, while not essential, this priming substantially increases colonization efficiency.^60^ As of yet, the MCP(s) involved in this behavior has not been identified, though determining which MCP(s) might be induced when chitobiose is present may provide a clue. Such a priming behavior has also been observed in the human gut microbiota; specifically, *Bacteroides thetaiotaomicron* responds to human-derived fucose residues by inducing enterocyte glycosylation.^83^

The study of the squid–vibrio model suggests another twist to the potential role of chemotaxis. Indeed, the squid light organ can be colonized either by “sharing” strains of *V. fischeri* that will co-exist with each other in the light-organ crypts, or by “dominant” strains that achieve a faster colonization and will typically mono-colonize a light organ. One hypothesis for this dominant behavior is that these strains have an enhanced or constitutive chemotaxis either toward chitibiose products, or toward other unknown chemoattractants to which only these strains are attracted.^2^

## Signaling the host via flagellar activity

Besides mediating motility, the flagella of some bacteria possess other functions, such as promoting invasion of host cells, biofilm formation or protein export.^84–87^ The latter has been recently discovered in the genus *Vibrio* for the first time as a mechanism by which *V. cholerae* secretes MakA, a cytotoxin in zebrafish and nematodes.^88^ However, in many models of pathogenesis, separating the role of bacterial motility from other such roles is difficult.^89^ In the squid–vibrio model, the initial events of a natural colonization can be observed in real-time, revealing the involvement of motility in both swimming,^51^ and host–bacteria communication.^4,6,51,90^

**Fig. 4:**
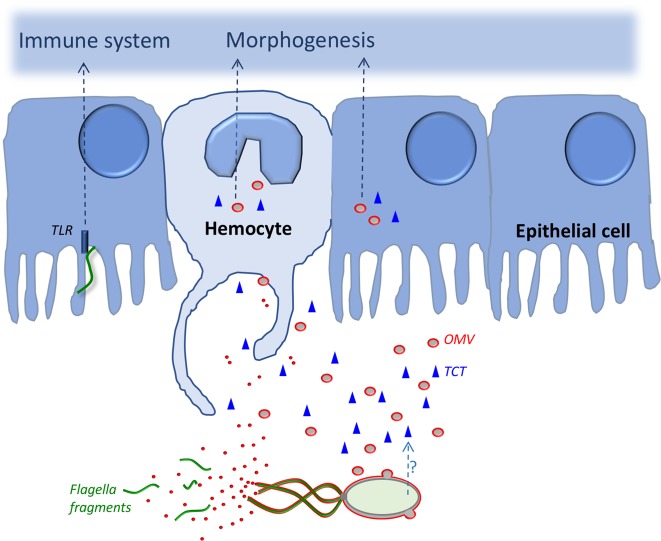
The flagellum as a platform for host–microbe interactions. Features of the flagellum and flagellar activity that contribute to host–symbiont interaction. In the squid host, epithelial cells and hemocytes can phagocytose OMVs (red) containing LPS and PGN (gray), which will, in turn, trigger steps in the host’s developmental morphogenesis. Soluble, released PGN monomer (TCT for Tracheal CytoToxin; blue) is also presented here, because it is an alternative form in which to present PGN fragments to the host, inducing morphogenesis. At the same time, squid homologs of TLRs may detect flagellins in fragments of the filament (green), and stimulate the immune system via the NF-κB pathway

The flagella of some bacterial species, such as *V. fischeri*, have an unusual sheath structure encasing them. Initially, this protective extension of the outer membrane was hypothesized to only serve to hide the immunogenic flagellins, limiting any host defensive response.^91^ More recently, studies of the squid–vibrio model have revealed an additional role: the rotation of the cell’s sheathed flagella releases outer membrane vesicles (OMVs)^90^ (Fig. 4). These OMVs contain, among other effectors, derivatives of two cell-surface molecules, peptidoglycan (PGN) and lipopolysaccharide (LPS),^8,51,90^ that are sufficient to trigger maturation of light-organ development.^92,93^ Because pathogenic species in the genera *Brucella, Helicobacter* and *Vibrio* also produce sheathed flagella,^94,95^ these structures may similarly play a role in the release of OMVs and subsequent host signaling, which perhaps facilitates their virulence.^96^ Finally, it remains a mystery how flagellar rotation leads to OMV release, and whether this blebbing originates from the flagellum’s tip, shaft and/or base, where the flagellar shaft passes through the outer membrane.^19,26,97^ Because the sheath appears to have a different composition than the cell’s outer membrane,^98,99^ we predict that the OMVs produced by the sheath may also have a different protein and/or lipid composition than those released from the bacterial cell body, and therefore may elicit distinct host responses.

The bacterial flagellar apparatus can directly participate in signaling the host in several ways. For instance, the apparatus is a type-3 secretion system (T3SS) and, in addition to flagellin subunits, it can be used to secrete virulence factors,^84,88,100–102^ which are injected into host cells, creating dramatic responses. Fewer examples have been reported of T3SS use in beneficial associations. In obligate intracellular mutualisms, the symbionts no longer need motility, and carriage of flagellar genes has been selected against during genome-size reduction. However, even in such symbioses, parts of the flagellar operon may be recruited to serve different purposes. Interestingly, only proteins involved in protein export within the flagellum-assembly pathway have been kept in some intracellular symbionts.^103^ For example, in the case the pea aphid and its endosymbiont *Buchnera aphidicola*, the symbionts are amotile, yet hundreds of flagellar basal bodies are assembled in the envelope of the bacterium. It has been suggested that these basal bodies could be used as a secretion system for proteins required to maintain the symbiosis.^104^

Another outcome of the loss of flagella for vertical transmitted, intracellular bacteria is that they avoid the possible activation of the host’s immune system by flagellin, which targets motile, extracellular bacteria. During bacterial interactions, the toll-like receptor-5 (TLR5) in mammalian-cell membranes acts in synergy with TLR4 to sense the presence of flagellin filaments, and activate the immune response,^89^ although flagellins within a sheath are essentially non-inflammatory.^91^ Thus, it is not surprising that the environmentally acquired symbionts that dominate vertebrate microbiota have mechanisms to suppress flagella expression after they reach their site of colonization. The presence of TLRs in some invertebrates suggests that their symbionts must also be mindful of similar immune responses. In the recently published genome sequence of *E. scolopes*, genes encoding TLR homologs have been identified,^105^ although none has been identified as a TLR5 homolog. While bacterial surface molecules induce the key phenotypes associated with light-organ maturation,^8^ flagellar fragments have only been shown to trigger persistence-associated changes in the host. Interestingly, a low abundance of some flagellar proteins can be detected within light-organ exudates of adult animals even though, when the bacteria reach the deep crypts of the light organ and proliferate to high density, they become aflagellate.^52,106^ These data suggest an as yet unknown role of these proteins in the host–symbiont communication (Fig. 4).

## Biofilm formation

After hatching, the aposymbiotic juvenile squids are exposed to diverse environmental bacteria, whose release of PGN stimulates the secretion of mucus onto the surface of the nascent light organs.^80,107^ Gram-negative marine bacteria present in the surrounding seawater become associated with the mucus and cilia, and form a biofilm-like aggregate of cells.^108^ When *V. fischeri* cells are present, they outcompete other bacteria in the aggregates.^80^ Within 3 h, as few as five aggregated *V. fischeri* cells will trigger a symbiont-specific transcriptional response in the host, before individually migrating toward the light-organ pores.^109^ However, beyond providing a platform for attachment, selection and priming,^81,109,110^ the roles of this aggregate are still not fully characterized, and certain strains of *V. fischeri* have been shown to produce extraordinarily large aggregates that can dominate other strains in competition experiments.^111^ Formation of this aggregate requires the bacterial *syp* locus, consisting of 18 genes involved in the synthesis and regulation of a polysaccharide biofilm matrix.^112,113^ Recent work has shown that NO, released by the host mucus,^114,115^ eventually down-regulates *syp*-gene expression^76^ and, thereby, may allow the bacteria to escape the aggregates and migrate to the pores.

Flagellar motility has long been described as an important factor for biofilm formation by different bacterial species;^116^ however, in the case of the squid–vibrio symbiosis, it appears that one role may be in dispersing cells from this biofilm. Indeed, while an aflagellate mutant is still capable of assembling an aggregate near the light organ’s pores, it is defective in dispersing from it and migrating into the ducts.^51^ Additionally, a hyperflagellated HS mutant assembles a delayed and smaller aggregate than the wild type, and colonizes more slowly, further indicating the importance of aggregate formation, perhaps for priming, prior to migration.^4^

Another aspect of flagellar motility involved in biofilm formation shown by in vitro studies of a number of bacterial species is that flagellar motility is important in the formation of a biofilm on abiotic surfaces. Indeed, an aflagellate mutant of *V. fischeri* on a glass surface produces only half of the amount of biofilm as its wild-type parent;^117^ however, an HS mutant produces a biofilm indistinguishable from the wild type.^4^ These results raise the question of what is it that brings bacteria together to form such aggregates if not flagellar motility? Recent work has provided evidence that the ciliary flow pattern around the nascent light organ selects and directs bacteria-sized particles into the aggregation region (Fig. 3c) adjacent to the pores.^29^ This study not only demonstrates the importance of host tissue in this complex and specific association, but also suggests that similar host-driven biomechanical events may be important in other beneficial (and pathogenic) symbioses. Finally, adhesion to host tissues is often a critical initiation step in bacterial colonization and persistence, both for pathogens and mutualists. For example, purified flagella of pathogenic *E. coli* can bind mucin.^118^ In contrast, in the squid–vibrio model, the flagellum itself does not seem to play a role in bacterial adhesion to the mucus-covered ciliated appendages because similar numbers of wild-type or aflagellated mutant cells typically attach;^51^ alternatively, perhaps in the case of *V. fischeri*, the presence of the sheath simply masks the adhesive flagellins. In fact, not only are flagella apparently not required for initial adhesion to the host, but also hyperflagellated HS cells adhere significantly less well than wild type.^4^ Taken together, analyses of many bacteria–host interactions indicate that the extensively studied role of the flagellar apparatus in biofilm formation is context-dependent.

## Conclusions and perspectives

Flagella are iconic appendages of bacteria, and are among the earliest morphological characters recognized by microbiologists. Although their functions have been well studied in *E. coli* and certain pathogenic bacteria, recent work has highlighted their no-less-essential role in establishing beneficial symbioses. For instance, the squid–vibrio model has emerged not only as a system in which to study the biology of this remarkably conserved cell structure, but also as one in which to discover the functions of atypical features, such as the presence of the flagellar sheath,^26^ the variation in flagellar number,^4^ or the newly reported flagellar wrapping behavior.^14,15,119^ This latter activity may provide a mechanism by which *V. fischeri* (and, by analogy, pathogenic *Vibrio* species) can use its flagella in a novel way to traverse highly confining viscous environments like the light-organ ducts and bottleneck,^120^ to reach their target tissues. It is worth noting that using flagellar motility as a mean to access a host is not specific to bacteria. For example, such motility also provides locomotion for a eukaryotic protist host. Such a relationship has been reported in two associations: the ectosymbiosis between a devescovinid flagellate and its protist host, *Caduceia versatilis*,^121^ and within the termite-gut microbiome, between *Mastotermes darwiniensis* and its ectosymbiont *Mixotricha paradoxa*.^122^

With all the genetic tools available in *V. fischeri*, the function of the entire flagellum structure can also be probed.^28^ For example, because this symbiont can assemble a polar tuft of up to 20 flagella,^4^ cryotomographic electron microscopy,^28^ aided by the high number of imageable structures per cell, could help create an understanding of how *V. fischeri* maintains its envelope integrity while extending its outer membrane to create individual flagellar sheaths.^26^ While cryotomographs of the H- and T-rings in *V. anguillarum* have been implicated in this extention, such imaging of the *V. fischeri* sheath-outer membrane junction reveals a different location for these rings.

More broadly, the importance of flagella and chemotaxis during colonization of a host, as well as during subsequent signaling between the partners, is now well recognized. However, what is less understood is the timing of when these functions are required during the initiation of a symbiosis. Using the squid–vibrio system, motility behavior and the presence of components of the assembled flagellum can be uncoupled, and the requirement for different flagellar functions be assayed at discrete times and tissue locations during the colonization process. Future investigations will introduce flagellated but non-motile *V. fischeri* cells directly into the crypts of the light organ, both to determine whether the flagella play a role in the persistence of the symbiosis, and to assay whether non-flagellated cells are preferentially lost. Thus, further studies of flagella in the squid–vibrio model will contribute both for an overall understanding of flagellar structure and diversity and for their ability to help us predict new functions present in other bacteria–host interactions.

### Reporting summary

Further information on research design is available in the Nature Research Reporting Summary linked to this article.

## Supplementary information

Reporting Summary Checklist
